# The clinical outcome of Dienogest treatment followed by in vitro fertilization and embryo transfer in infertile women with endometriosis

**DOI:** 10.1186/s13048-019-0597-y

**Published:** 2019-12-12

**Authors:** Hiroshi Tamura, Hiroaki Yoshida, Hiroyuki Kikuchi, Mai Josaki, Yumiko Mihara, Yuichro Shirafuta, Masahiro Shinagawa, Isao Tamura, Toshiaki Taketani, Akihisa Takasaki, Norihiro Sugino

**Affiliations:** 1 Department of Obstetrics and Gynecology, Yamaguchi University Graduate School of Medicine, Minamikogushi 1-1-1, Ube, 755-8505 Japan; 2 Center for Reproductive Medicine, Sendai ART Clinic, Nakakecho 206-13, Miyagino-ku, Sendai, 983-0864 Japan; 3 Department of Obstetrics and Gynecology, Saiseikai Shimonoseki General Hospital, Yasuokacho 8-5-1, Shimonoseki, 759-6603 Japan

**Keywords:** Endometriosis, Dienogest, Infertile, IVF-ET, Growing follicle

## Abstract

**Background:**

Endometriosis is considered to be the most intractable cause of female infertility. Administering any type of treatment for endometriosis before in vitro fertilization and embryo transfer (IVF-ET) is an important strategy for improving the IVF-ET outcomes for infertile women with endometriosis. In fact, treatment with a gonadotropin-releasing hormone (GnRH) agonist just before IVF-ET has been reported to improve the clinical outcome in endometriosis patients. However, the benefit of Dienogest (DNG), a synthetic progestin, treatment just before IVF-ET remains unclear.

**Methods:**

Sixty-eight infertile women with Stage III or IV endometriosis (ovarian endometrial cyst < 4 cm) were recruited for this study. The subjects were divided into 2 groups: a DNG group (*n* = 33) and a control group (*n* = 35). DNG was administered orally every day for 12 weeks prior to the conventional IVF-ET cycle in the DNG group. Standard controlled ovarian hyperstimulation with the GnRH agonist long protocol was performed in the control group. The numbers of mature follicles and retrieved oocytes, fertilization rates, implantation rates, and clinical pregnancy rate were compared between the two groups. In addition, the concentrations of inflammatory cytokines, oxidative stress markers, and antioxidants in follicular fluids were also measured.

**Results:**

The numbers of growing follicles, retrieved oocytes, fertilized oocytes, and blastocysts were significantly lower in the DNG group than in the control group. The fertilization and blastocyst rates were also lower in the DNG group than in the control group. Although there was no significant difference in the implantation rate between the groups, the cumulative pregnancy rate and live birth rate were lower in the DNG group than in the control group. There was no significant difference in the abortion rate. Our results failed to show that DNG reduces the inflammatory cytokine levels and oxidative stress in follicular fluids.

**Conclusions:**

Administering DNG treatment just before IVF-ET did not provide any benefits to improve the clinical outcomes for infertile women with endometriosis.

## Introduction

Endometriosis is characterized by the presence of endometrial-like tissue (glands and stroma) outside the uterus and affects up to 15% of reproductive-age women [1]. It can be found in up to 50% of infertile women [2], and patients with endometriosis suffer from infertility in about 30 to 50% of cases [3], suggesting a deep relationship between endometriosis and infertility. Infertile women with endometriosis often require assisted reproduction technology (ART), such as in vitro fertilization and embryo transfer (IVF-ET) [4], to become pregnant; however, the success rate of IVF-ET in these patients is almost half that in women without endometriosis [5, 6]. The exact mechanisms underlying endometriosis-associated infertility remain unclear. However, multiple hypotheses have been suggested to explain the low fecundity observed with endometriosis, including a distorted tubo-ovarian anatomy [7], induction of inflammation [8–10], oxidative damage [11, 12], and a poor oocyte quality [13]. A better understanding of the pathogenesis of endometriosis-associated infertility is crucial for improving infertility treatment outcomes. Therefore, the management of endometriosis-associated infertility to improve the ART outcome is a major issue in reproductive medicine.

Although an improvement in the pregnancy rate has been reported upon removing endometriosis lesions and adhesiolysis under laparoscopy [14, 15], the mechanical removal of endometriomas impairs the ovarian reserve, leading to a reduced number of follicles and oocytes [16–18]. In addition, the European Society of Human Reproduction and Embryology (ESHRE) guideline found no evidence to support the recommendation to perform surgical excision of deep nodular lesions prior to ART in order to improve reproductive outcomes [19, 20].

One approach to optimizing ART outcomes in infertile women with endometriosis is prolonged pre-cycle suppressive hormone therapy. Down-regulation for 3 to 6 months with a gonadotropin-releasing hormone (GnRH) agonist in women with endometriosis increases the odds of clinical pregnancy four-fold [21]. We recently reported that the ultralong protocol using a GnRH agonist for 3 months prior to IVF-ET improves the reproductive outcomes by reducing the detrimental effects of cytotoxic cytokines and oxidative stress in infertile women with endometriosis [22].

Dienogest (DNG) is a fourth-generation progestin, a derivative of 19-norsteroids, highly selective for the progesterone receptor agonist. DNG has direct growth inhibitory action on endometriosis lesions and inhibitory action on cytokines, such as interleukin-8 (IL-8), and reportedly has stronger cytoreductive effects on endometriosis lesions than GnRH agonist [23–25]. Therefore, the use of DNG prior to IVF-ET may further improve the implantation rate and pregnancy rate of IVF-ET.

In the present study, we administered DNG prior to IVF-ET for infertility associated with endometriosis to examine whether or not the clinical outcome was improved. To elucidate the mechanism by which DNG improves the IVF-ET outcomes, the concentrations of inflammatory cytokines, oxidative stress markers, and antioxidants in follicular fluids were also analyzed.

## Materials and method

This study was a prospective randomized control study of infertile women with endometriosis who planned to undergo IVF-ET at Yamaguchi University School of Medicine, Yoshida Ladies’ Clinic, and Saiseikai Shimonoseki General Hospital between February 2011 and November 2015 (Clinical Trial. ID: UMIN 000005234).

The study inclusion criteria were as follows: infertile women 20 to 40 years of age with endometrial ovarian cysts (< 4 cm) diagnosed by ultrasonography or MRI or laparoscopy. The stage of endometriosis (stage III or IV) was defined by the revised American Society for Reproduction Medicine (rASRM) classification via laparoscopy. Women using hormonal contraceptives or other hormonal therapies or who had a disease condition that might interfere with the conduct of the study were excluded. A total of 68 women were therefore included in this study.

The subjects were randomized to either the DNG group or control group using the envelope method with envelopes distributed by the Department of Obstetrics and Gynecology at Yamaguchi University School of Medicine to each facility (DNG group: 33 cases, control group: 35 cases).

The study protocol was reviewed and approved by Ethics Committee/Institutional Review Board of Yamaguchi University School of Medicine (H22–165), and the study was conducted according to guidelines described in the Declaration of Helsinki. Written informed consent was provided by all subjects prior to study inclusion.

### Study design

**Fig. 1:**
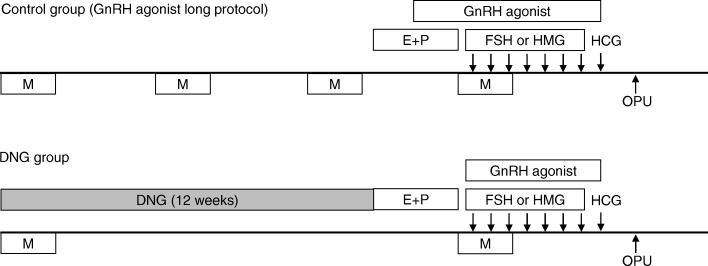
Schematic representation of the study protocol for the DNG group and control group In the control group, patients received standard controlled ovarian hyperstimulation with mid-luteal phase GnRH agonist down-regulation. Nasal spray GnRH agonist was administered from the mid-luteal phase in the previous cycle to the time of HCG injection for the ovulation induction of the IVF-ET cycle. Controlled ovarian hyperstimulation was initiated from the second day of the IVF-ET cycle by FSH and HMG. In the DNG group, DNG was administered orally every day for 12 weeks from 3 months prior to the IVF-ET cycle. Withdrawal bleeding was induced using E and P. Controlled ovarian hyperstimulation was given in a manner similar to that in the control group. GnRH agonist: buserelin acetate 900 mg/day; E: conjugated estrogen; P: dydrogesterone; M: menstruation; FSH: follicle-stimulating hormone; HMG: human menopausal gonadotropin; HCG: human chorionic gonadotropin; OPU: ovum pick-up; DNG: dienogest; dinagest, 2 mg/day.

Figure 1 is a schematic summary of the study protocol. In the DNG group, DNG (dinagest, 2 mg/day; Mochida Pharmaceutical Co. Ltd., Tokyo, Japan) was administered orally every day for 12 weeks from 3 months prior to the IVF-ET cycle. Withdrawal bleeding was induced using estrogen (Premarin 0.625 mg: conjugated estrogen tablets, 2 tablets/day for 21 days; Pfizer Pharmaceutical Co., Tokyo, Japan) and progesterone (Duphaston 5 mg: dydrogesterone tablets, 3 tablets for 12 days; Daiichi-Sankyo Co., Ltd., Tokyo, Japan) before controlled ovarian hyperstimulation. Controlled ovarian hyperstimulation was initiated from the second day of the IVF-ET cycle by injecting 225 IU follicle-stimulating hormone (FSH; Folyrmon P; Fuji Pharmaceutical Co., Ltd., Tokyo, Japan) for 3 days, followed by a daily injection of 150 IU human menopausal gonadotropin (HMG; HMG F; Fuji Pharmaceutical Co., Ltd.). Nasal spray GnRH agonist (900 μg/day buserelin acetate, Suprecure; Mochida Pharmaceutical) was also given from the second day of the IVF-ET cycle to continuously suppress pituitary gonadotropin secretion until the injection of human chorionic gonadotropin (HCG; HCG Mochida 10,000 IU; Mochida Pharmaceutical) for ovulation induction.

In the control group, patients received standard controlled ovarian hyperstimulation with mid-luteal phase GnRH agonist down-regulation (long protocol). Nasal spray GnRH agonist (900 μg/day buserelin acetate, Suprecure; Mochida Pharmaceutical) was administered from the mid-luteal phase in the previous cycle to the time of HCG injection for the ovulation induction of the IVF-ET cycle. Controlled ovarian hyperstimulation was given in a manner similar to that in the DNG group described above.

When leading follicles reached ≥18 mm in diameter, HCG was injected for ovulation induction. Oocyte retrieval was carried out 35 h after HCG injection, with follicular fluids collected from mature follicles (≥18 mm) aspirated separately in the left and right ovaries (one specimen from each side). The follicular fluids containing the oocyte were collected, and immediately after removal of the oocyte, each of the follicular fluid samples was centrifuged at 300 *g* for 15 min to remove cellular components. The supernatant from each follicle was mixed for the same patient and kept at − 80 °C until assayed.

The numbers of mature follicles, retrieved oocytes, and fertilized oocytes as well as the fertilization rates, implantation rates, and clinical pregnancy rates were compared between the two groups. Concentrations of inflammatory cytokines (tumor necrosis factor alpha [TNF-α], interleukin-6 [IL-6], IL-8) and oxidative stress markers (8-hydroxy-2′-deoxyguanosine [8-OHdG] as a marker of DNA damage; hexanoyl-lysine adduct [HEL] as a marker of lipid peroxidation) as well as antioxidants (Cu,Zu-superoxide dismutase [Cu,Zn-SOD], melatonin) in follicular fluids were measured using an enzyme-linked immunosorbent assay (ELISA) kit as described below.

Retrieved cumulus–oocyte complexes were fertilized either by conventional insemination or by intracytoplasmatic sperm injection based on the sperm quality. Embryo transfer to the uterine cavity was carried out on day 3 or 5 of in vitro cultivation. Embryos remaining after embryo transfer were stored at a low temperature using the vitrification technique for future use. In the subsequent cycle, the vitrified embryos were warmed and thawed before being transferred into the uterus after preparation of the endometrium with transdermal estradiol and vaginal progesterone. The stage of endometriosis (r-ASRM classification: cases undergoing laparoscopy), serum CA 125 levels, FSH (HMG) dosage, serum estradiol and progesterone levels, number of growing follicles (follicular diameter ≥ 15 mm), number of mature follicles, number of retrieved oocytes, number of fertilized oocytes, and number of blastocysts were evaluated. Clinical pregnancy was established on confirmation of the gestational sac by transvaginal ultrasonography.

### The measurement of cytokines, oxidative stress markers, and antioxidants in follicular fluid

The concentration of TNF-α in follicular fluid was measured using an ELISA kit for TNF-α (Human TNF-α ELISA kit, HSTA00D; BIO-TECHNE, Minneapolis, MN, USA) and the concentrations of IL-6, and IL-8 were measured using ELISA kit IL-6 (Human IL-6 ELISA kit, EH2IL65; Human IL-8 ELISA kit, EH2IL85, Thermo Fisher Scientific Pierce Biotechnology, Rockford, IL, USA). Each sample of follicular fluid (50 μl) was used for duplicate assay according to the assay protocol. The sensitivity of TNF-α was 0.106 pg/ml, and the intra- and inter-assay coefficients of variation (CVs) were 4.5 and 5.2%, respectively. The sensitivity of IL-6 was 1 pg/ml, and the intra- and inter-assay CVs were both < 10%. The sensitivity of IL-8 was 2 pg/ml, and the intra- and inter-assay CVs were both < 10%.

The 8-OHdG concentrations were measured using a New 8-OHdG Check ELISA kit (High sensitivity 8-OHdG measurement kit, KOG-HS10/E; Japan Institute for the Control of Aging, Nikken SEIL Co., Ltd., Shizuoka, Japan) as we reported previously [26, 27]. Each sample of follicular fluid (50 μl) filtered using an ultrafilter (cut-off molecular weight: 10 kDa) was used for duplicate assays. The sensitivity of 8-OHdG was 0.125 ng/ml, and the intra- and inter-assay CVs were 5.5 and 6.1%, respectively.

The HEL concentrations were measured using an ELISA kit (hexanoyl lysine measurement kit, KHL-700; Japan Institute for the Control of Aging) as reported previously [26, 27]. Each sample of follicular fluid (50 μl) was pretreated with chymotrypsin to perform proteolysis and then filtered using an ultrafilter (cut-off molecular weight: 10 kDa) for duplicate assays. The minimal detectable concentration of HEL was estimated to be 2 nmol/L, and the intra- and inter-assay CVs were both < 10%.

The Cu,Zn-SOD concentrations were measured using a Human Cu/Zn-superoxide dismutase ELISA kit (human Cu/Zn-SOD ELISA kit, KT-034; Northwest Life Science Specialties, LLC, Vancouver, WA, USA) as reported previously [26, 27]. Each sample of follicular fluid (20 μl) was used for duplicate assays, according to the assay protocol. The sensitivity of Cu,Zn-SOD was 0.04 ng/ml, and the intra- and inter-assay CVs were 5.1 and 5.8%, respectively.

The melatonin concentrations were measured using an ELISA kit (Human Melatonin, MT ELISA kit, KT-21916; Kamiya Biomedical Company, Seattle, WA, USA). Each sample of follicular fluid (50 μl) was used for duplicate assays, according to the assay protocol. The sensitivity of the assay was 1 pg/ml, and the intra- and inter-assay CVs were both < 10%.

### Statistical analyses

Statistical analyses were carried out with the SPSS software program for Windows, ver. 13.0 (SPSS Inc., Chicago, IL, USA). The Mann-Whitney U-test using Bonferroni’s correction and Fisher’s test were employed as appropriate. Correlations were analyzed using Spearman’s rank correlation coefficient. Differences were considered to be significant if *P* < 0.05.

## Results

**Fig. 2:**
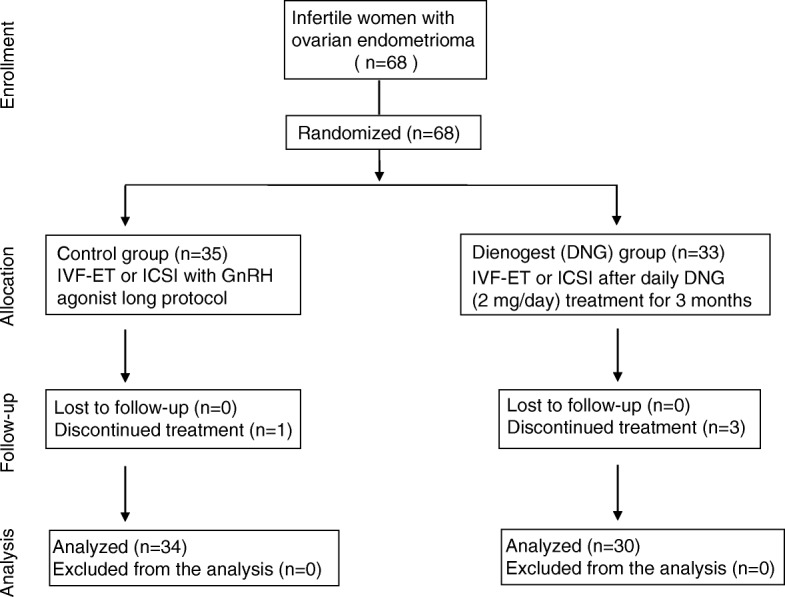
CONSORT statement flow diagram

Figure 2 shows the flow of participants through the study. A total of 68 women were randomized (control group, *n* = 35; DNG group, *n* = 33). Four women (control group, *n* = 1; DNG group, *n* = 3) were excluded from this study due to their discontinuing fertility treatment. The remaining 64 participants (control group, *n* = 34; DNG group, *n* = 30) were analyzed. There was no marked difference in the age between the 2 groups (DNG group: 34.2 ± 3.4 years; control group: 33.6 ± 3.6 years), nor in the stage of endometriosis evaluated according to the r-ASRM classification by laparoscopy or in the serum CA 125 levels (DNG group: 29.1 ± 26.9 U/ml; control group: 26.4 ± 16.8 U/ml) (Table 1). The total amount of FSH/HMG used for follicle stimulation (DNG group: 1553 ± 528 IU; control group: 1304 ± 338 IU) was greater in the DNG group than in the control group, while the number of growing follicles (DNG group: 4.6 ± 3.2; control group: 6.5 ± 4.2) and the serum E2 levels (DNG group: 1288 ± 1091 pg/ml; control group: 1731 ± 958 pg/ml) were significantly lower in the DNG group than in the control group (Table 1).

**Table 1:** Clinical characteristics and IVF-ET data

	Control	DNG	p
Patients (n)	34	30	
Age (years)	33.6 ± 3.6	34.2 ± 3.4	ns
Stage of endometriosis rASRM (III)	9	10	
rASRM (IV)	5	6	
Serum CA125 (U/ml) (before DNG)	–	44.2 ± 35.3	
Serum CA125 (U/ml)	26.4 ± 16.8	29.1 ± 26.9	ns
Gonadotropin (FSH/HMG) dose (IU)	1304 ± 338	1553 ± 528	P < 0.05
Estradiol (pg/ml)	1731 ± 958	1288 ± 1091	P < 0.05
Progesterone (ng/ml)	1.11 ± 1.14	1.72 ± 2.0	ns
Follicles ≥15 mm (n)	6.5 ± 4.2	4.6 ± 3.2	P < 0.05

*IVF-ET* In vitro fertilization and embryo transfer, *DNG* Dienogest

The number of retrieved oocytes (DNG group: 5.0 ± 3.6; control group: 7.5 ± 4.2) and growing follicles (DNG group: 4.1 ± 3.1; control group: 6.6 ± 3.9) were significantly lower in the DNG group than in the control group (Table 2). The number of fertilized oocytes (DNG group: 2.8 ± 2.8; control group: 5.0 ± 3.2) and fertilization rate (DNG group: 53.7% ± 33.0%; control group: 68.0% ± 23.4%) were significantly lower in the DNG group than in the control group (Table 2). The number of blastocysts (DNG group: 1.5 ± 1.7; control group: 3.9 ± 2.9) was also significantly lower in the DNG group than in the control group (Table 2). Although there was no significant difference between the groups in the implantation rate (DNG group: 25.0%, 11/44; control group: 35.3%, 24/68), the cumulative pregnancy rate (DNG group: 33.7%, 11/30; control group: 67.6%, 23/34) and live birth rate (DNG group: 23.3%, 7/30; control group: 52.9%, 18/34) were lower in the DNG group than in the control group. There was no significant difference in the abortion rate (DNG group: 36.4%, 4/11; control group: 25.0%, 6/24) between the 2 groups (Table 2).

**Table 2:** IVF-ET data and clinical outcomes

	Control (*n* = 34)	DNG (*n* = 30)	*p*
Oocytes retrieved (n)	7.5 ± 4.2	5.0 ± 3.6	*P* < 0.01
Mature oocytes (n)	6.6 ± 3.9	4.1 ± 3.1	*P* < 0.01
Fertilized oocytes (n)	5.0 ± 3.2	2.8 ± 2.8	*P* < 0.01
Fertilization rate (%)	68.0 ± 23.4	53.7 ± 33.0	*P* < 0.01
Blastocysts (n)	3.9 ± 2.9	1.5 ± 1.7	*P* < 0.01
Embryos transferred (n)	68	44	
Gestational sacs (n)	24	11	
Implantation rate (%)	35.3 (24/68)	25.0 (11/44)	ns
Pregnancy rate (%)	67.6 (23/34)	33.7 (11/30)	*P* < 0.05
Live birth rate (%)	52.9 (18/34)	23.3 (7/30)	*P* < 0.05
Abortion rate (%)	25.0 (6/24)	36.4 (4/11)	ns

*IVF-ET* in vitro fertilization and embryo transfer, *DNG* dienogest, *rASRM* revised American society for reproduction medicine, *FSH* follicle-stimulating hormone, *HMG* human menopausal gonadotropin

**Fig. 3:**
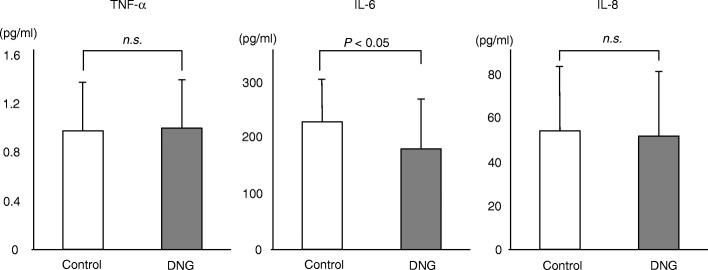
Concentrations of inflammatory cytokines in follicular fluids Thirty patients received 12 weeks of Dienogest treatment (2 mg/day) followed by standard controlled ovarian hyperstimulation (COH) for IVF-ET (DNG group). Thirty-four patients received standard COH with mid-luteal phase GnRH agonist down-regulation (control group). The concentrations of inflammatory cytokines, including tumor necrosis factor alpha (TNF-α), interleukin-6 (IL-6), and interleukin-8 (IL-8), were measured in the follicular fluid obtained at the time of oocyte retrieval. Values are the mean ± SD. Statistical analyses were performed with the Mann-Whitney U-test using Bonferroni’s correction.

**Fig. 4:**
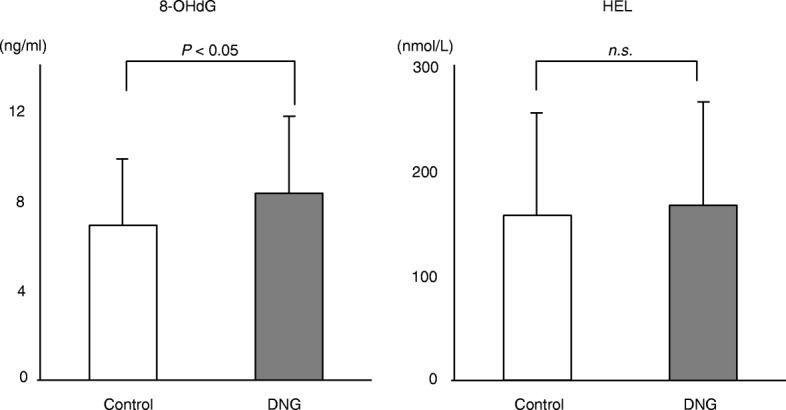
Concentrations of oxidative stress markers in follicular fluids Thirty patients received 12 weeks of Dienogest treatment (2 mg/day) followed by standard controlled ovarian hyperstimulation (COH) for IVF-ET (DNG group). Thirty-four patients received standard COH with mid-luteal phase GnRH agonist down-regulation (control group). The concentrations of the oxidative stress markers 8-hydroxy-2′-deoxyguanosine (8-OHdG) as a marker of DNA damage and hexanoyl-lysine adduct (HEL) as a marker of lipid peroxidation, were measured in the follicular fluid obtained at the time of oocyte retrieval. Values are the mean ± SD. Statistical analyses were performed with the Mann-Whitney U-test using Bonferroni’s correction.

**Fig. 5:**
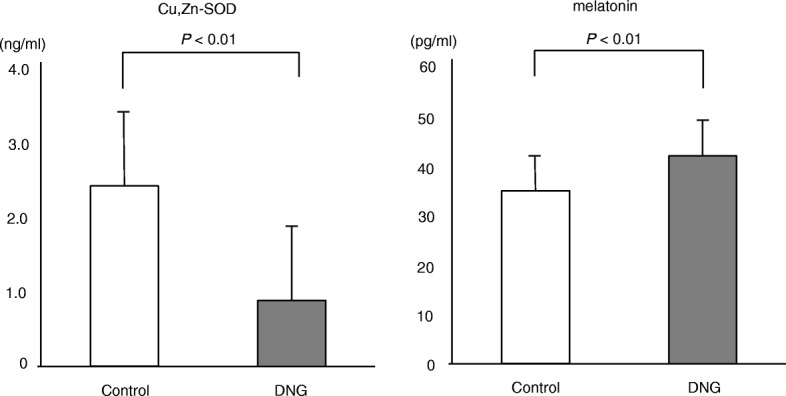
Concentrations of antioxidants in follicular fluids Thirty patients received 12 weeks of Dienogest treatment (2 mg/day) followed by standard controlled ovarian hyperstimulation (COH) for IVF-ET (DNG group). Thirty-four patients received standard COH with mid-luteal phase GnRH agonist down-regulation (control group). The concentrations of the antioxidants Cu,Zu-superoxide dismutase (Cu,Zn-SOD) and melatonin were measured in the follicular fluid obtained at the time of oocyte retrieval. Values are the mean ± SD. Statistical analyses were performed with the Mann-Whitney U-test using Bonferroni’s correction.

Follicular fluids from mature follicles (≥18 mm) in the left and right ovaries were aspirated separately and were sampled for the analysis. The average value of both follicles was taken as the value for the patient. With regard to inflammatory cytokines, while no marked difference between the groups was noted in the TNF-α (DNG group: 0.98 ± 0.43 pg/ml; control group: 0.97 ± 0.35 pg/ml) or IL-8 levels (DNG group: 51.9 ± 33.9 pg/ml; control group: 53.8 ± 28.8 pg/ml), the IL-6 level (DNG group: 179 ± 102 pg/ml; control group: 229 ± 66 pg/ml) was lower in the DNG group than in the control group (Fig. 3). With regard to oxidant stress markers in the follicular fluid, while the level of 8-OHdG (DNG group: 8.2 ± 3.9 ng/ml; control group: 6.4 ± 2.9 ng/ml) was higher in the DNG group than in the control group, no marked difference between the groups was noted in the lipid peroxidation marker HEL (DNG group: 168 ± 102 nmol/L; control group: 158 ± 104 nmol/L) (Fig. 4). While the antioxidant enzyme Cu/Zn-SOD (DNG group: 0.89 ± 0.55 ng/ml; control group: 2.43 ± 2.16 ng/ml) was lower in the DNG group than in the control group, the melatonin concentration (DNG group: 42.5 ± 7.0 pg/ml; control group: 35.4 ± 7.1 pg/ml) was higher in the DNG group than in the control group (Fig. 5).

## Discussion

Regarding approaches to optimizing IVF-ET outcomes in infertile women with endometriosis, the use of prolonged GnRH agonist treatment (for 3 to 6 months) prior to IVF-ET has been reported to improve the pregnancy rate [21, 22, 28]. Indeed, we demonstrated that the ultralong protocol using a GnRH agonist for 3 months prior to IVF-ET improved the reproductive outcomes by reducing the detrimental effects of cytotoxic cytokines and oxidative stress in infertile women with endometriosis [22]. We therefore expected the improving effect of DNG on the IVF-ET clinical outcomes as well as GnRH agonist by shrinking endometriosis lesions and reducing inflammatory cytokine levels and oxidant stress.

However, our results showed that DNG treatment just before IVF-ET did not provide any benefits to improve IVF-ET outcomes in infertile women with endometriosis. We found that treatment with DNG prior to IVF-ET in infertile women with endometriosis reduced the number of growing follicles, retrieved oocytes, fertilized oocytes, and blastocysts, resulting in a decline in the pregnancy rate. The decrease in the number of growing follicles in the DNG group was considered to be a major cause of the decline in the pregnancy rate. The reason for the decreased number of growing follicles by treatment with DNG prior to IVF-ET is unclear, but the action of DNG on the follicles may be involved. Suppression of follicle growth and induction of follicle atresia are known effects of progestins [29, 30], and DNG also has the same effect [31, 32]. The use of DNG prior to IVF-ET may therefore have resulted in the suppression of follicle growth and induction of follicle atresia. The ovarian stimulation immediately after DNG treatment may have reduced the number of growing follicles, because of the small number of antral follicles capable of responding to FSH/HMG stimulation. A certain period of time may be necessary in order to increase the number of antral follicles capable of responding to FSH/HMG after DNG treatment before starting the IVF-ET program. Another potential mechanism is that DNG inhibits the recruitment of primordial follicles into growing follicles [33]. In addition to suppressing follicular growth and inducing follicular atresia, reducing the recruitment of primordial follicles may also contribute to the decrease in the number of growing follicles by DNG.

Accumulating evidence has shown that DNG reduces endometriosis lesions, resulting in a reduction in inflammatory cytokines [25, 34], which may lead to improved IVF-ET outcomes. TNF-α is significantly increased in the peritoneal fluid of women with endometriosis [35] and has been associated with endometriosis progression and related infertility [36]. TNF-α can also stimulate the production of other pro-inflammatory cytokines, including IL-6 and IL-8, in the stromal cells through the regulation of the nuclear factor kappa B (NF-κB) activation. Our previous study showed that the concentrations of TNF-α, a cytotoxic cytokine, in follicular fluids were significantly lower in the ultralong GnRH agonist therapy group than in the control group [22]. In contrast, the level of TNF-α in follicular fluid was not reduced by 3 months of DNG treatment in this study. The mechanism underlying the anti-inflammatory effect of DNG in endometrial lesions is still obscure, since most of the evidence comes from in vitro studies using endometrial stromal cells [25, 34, 37]. On the other hand, it has been reported that DNG is able to reduce the size of endometriotic lesions [24, 38]. It may be possible that the production of pro-inflammatory cytokines is decreased as a consequence of the reduced size of endometrial lesions following DNG treatment. Most of these clinical data on DNG reflect follow-up durations of 6 months or longer [39–41]. Three months of DNG treatment in this study may therefore have been insufficient for achieving a suitable reduction in the endometrial cyst size.

Oxidative stress caused by reactive oxygen species (ROS) plays an important role in the pathogenesis of endometriosis. Increased numbers of erythrocytes in women with endometriosis releases heme and iron into the peritoneal environment. ROS production due to iron overload induces an increase in NF-κB in peritoneal macrophages, leading to pro-inflammatory, growth, and angiogenic factors in women with endometriosis [42–44]. The correlation between oxidative stress and the oocyte quality in infertile women with endometriosis has received increasing attention [42, 45]. Several studies have shown that the follicular fluid of women with endometriosis had increased levels of ROS and a reduced total antioxidant capacity [46–48]. Our previous study showed that ultralong GnRH agonist therapy reduced the concentrations of oxidative stress (8-OHdG) in the follicular fluids of infertile women with endometriosis [22]. However, the level of 8-OHdG in the follicular fluid was not reduced by 3 months of DNG treatment in the present study. At present, there is no clear evidence that DNG reduces oxidative stress in follicular fluids and peritoneal fluids in women with endometriosis. Further studies are necessary to clarify the involvement of DNG in the oxidative status of the peritoneal cavity in infertile women with endometriosis.

In the present study, we used conjugated estrogen and progestin to induce withdrawal bleeding before controlled ovarian hyperstimulation in both DNG group and control group, because the same induction protocol was necessary to compare the outcomes of IVF-ET between two groups. However, there is a possibility that estrogen treatment has some influence on the endometriosis lesions. Further studies using alternative protocol of DNG treatment before IVF-ET without using estrogen agents are necessary to evaluate the more accurate effect of DNG treatment on IVF-ET outcomes in infertile women with endometriosis.

## Conclusion

In the present study, the use of DNG prior to IVF-ET did not improve the clinical outcomes. This finding may be attributed to mechanisms such as the suppression of follicle growth and induction of follicular atresia of progesterone. Ovarian stimulation was started immediately after DNG treatment with this IVF-ET protocol. Some interval between DNG treatment and ovarian stimulation may be necessary to allow small follicles to grow into antral follicles capable of responding to FSH stimulation, thereby resulting in an increase in the number of growing follicles.

## Data Availability

The datasets used and/or analyzed during the current study are available from the corresponding author on reasonable request.

## Abbreviations

8-OHdG: 8-hydroxy-2′-deoxyguanosine
ART: assisted reproduction technology
Cu,Zn-SOD: Cu,Zu-superoxide dismutase
DNG: dienogest
FSH: follicle-stimulating hormone
GnRH: gonadotropin-releasing hormone
HCG: human chorionic gonadotropin
HEL: hexanoyl-lysine adduct
HMG: human menopausal gonadotropin
IL-6: interleukin-6
IL-8: interleukin-8
IVF-ET: in vitro fertilization and embryo transfer
TNF-α: tumor necrosis factor alpha
